# Peroxisome proliferator-activated receptor ɣ agonist mediated inhibition of heparanase expression reduces proteinuria

**DOI:** 10.1016/j.ebiom.2023.104506

**Published:** 2023-03-06

**Authors:** Marjolein Garsen, Baranca Buijsers, Marloes Sol, Lena Gockeln, Ramon Sonneveld, Toin H. van Kuppevelt, Mark de Graaf, Jacob van den Born, Jan A.A.M. Kamps, Daniël H. van Raalte, Rutger W. van der Meer, Hildo J. Lamb, Jan-Luuk Hillebrands, Ton J. Rabelink, Marissa L. Maciej-Hulme, Guido Krenning, Tom Nijenhuis, Johan van der Vlag

**Affiliations:** a Department of Nephrology, Radboud Institute for Molecular Life Sciences, Radboud University Medical Center, Nijmegen, the Netherlands; b Division of Medical Biology, Department of Pathology and Medical Biology, University Medical Center Groningen, Groningen, the Netherlands; c Department of Biochemistry, Radboud Institute for Molecular Life Sciences, Radboud University Medical Center, Nijmegen, the Netherlands; d Division of Nephrology, Department of Internal Medicine, University Medical Center Groningen, Groningen, the Netherlands; e Diabetes Center, VU University Medical Center, Amsterdam, the Netherlands; f Department of Radiology, Leiden University Medical Center, Leiden, the Netherlands; g Division of Pathology, University Medical Center Groningen, Groningen, the Netherlands; h Department of Nephrology and Einthoven Laboratory for Vascular Medicine, Leiden University Medical Center, Leiden, the Netherlands

**Keywords:** Peroxisome proliferator-activated receptor ɣ, Heparanase, Proteinuria, Thiazolidinediones, Glomerular endothelial cells, Podocytes

## Abstract

**Background:**

Proteinuria is associated with many glomerular diseases and a risk factor for the progression to renal failure. We previously showed that heparanase (HPSE) is essential for the development of proteinuria, whereas peroxisome proliferator-activated receptor ɣ (PPARɣ) agonists can ameliorate proteinuria. Since a recent study showed that PPARɣ regulates HPSE expression in liver cancer cells, we hypothesized that PPARɣ agonists exert their reno-protective effect by inhibiting glomerular HPSE expression.

**Methods:**

Regulation of HPSE by PPARɣ was assessed in the adriamycin nephropathy rat model, and cultured glomerular endothelial cells and podocytes. Analyses included immunofluorescence staining, real-time PCR, heparanase activity assay and transendothelial albumin passage assay. Direct binding of PPARɣ to the HPSE promoter was evaluated by the luciferase reporter assay and chromatin immunoprecipitation assay. Furthermore, HPSE activity was assessed in 38 type 2 diabetes mellitus (T2DM) patients before and after 16/24 weeks treatment with the PPARɣ agonist pioglitazone.

**Findings:**

Adriamycin-exposed rats developed proteinuria, an increased cortical HPSE and decreased heparan sulfate (HS) expression, which was ameliorated by treatment with pioglitazone. In line, the PPARɣ antagonist GW9662 increased cortical HPSE and decreased HS expression, accompanied with proteinuria in healthy rats, as previously shown. *In vitro*, GW9662 induced HPSE expression in both endothelial cells and podocytes, and increased transendothelial albumin passage in a HPSE-dependent manner. Pioglitazone normalized HPSE expression in adriamycin-injured human endothelial cells and mouse podocytes, and adriamycin-induced transendothelial albumin passage was reduced as well. Importantly, we demonstrated a regulatory effect of PPARɣ on HPSE promoter activity and direct PPARy binding to the HPSE promoter region. Plasma HPSE activity of T2DM patients treated with pioglitazone for 16/24 weeks was related to their hemoglobin A1c and showed a moderate, near significant correlation with plasma creatinine levels.

**Interpretation:**

PPARɣ-mediated regulation of HPSE expression appears an additional mechanism explaining the anti-proteinuric and renoprotective effects of thiazolidinediones in clinical practice.

**Funding:**

This study was financially supported by the 10.13039/501100002997Dutch Kidney Foundation, by grants 15OI36, 13OKS023 and 15OP13. Consortium grant LSHM16058-SGF (GLYCOTREAT; a collaboration project financed by the PPP allowance made available by 10.13039/100016036Top Sector Life Sciences & Health to the 10.13039/501100002997Dutch Kidney Foundation to stimulate public-private partnerships).

**Research in context**

**Evidence before this study**

The heparan sulfate-degrading enzyme, heparanase, is essential for the degradation of glomerular endothelial glycocalyx and the development of proteinuria in glomerular diseases. Thiazolidinediones are synthetic, high affinity agonists for the nuclear transcription factor, peroxisome proliferator-activated receptor ɣ (PPARɣ), which ameliorates proteinuria in certain glomerular diseases.

**Added value of this study**

This paper describes that heparanase is regulated by PPARy both *in vitro*, in cultured glomerular cells, and *in vivo,* in a rat model for focal segmental glomerulosclerosis. In humans treated with pioglitazone, plasma heparanase activity tended to be decreased. It is demonstrated that PPARɣ directly binds to the heparanase promotor region in both podocytes and glomerular endothelial cells.

**Implications of all the available evidence**

PPARy-mediated regulation of heparanase expression provides an additional mechanism that explains the anti-proteinuric and renoprotective effects of thiazolidinediones in clinical practice.

## Introduction

Proteinuria is one of the first clinical signs of many glomerular diseases and an independent risk factor for the progression to renal failure.^1^ Proteinuria can be caused by damage to any of the 3 layers of the glomerular filtration barrier (GFB), which is composed of a fenestrated endothelium covered with a glycocalyx, the glomerular basement membrane and podocytes. All layers of the GFB should be intact to prevent the development of proteinuria.^2^

Peroxisome proliferator-activated receptor ɣ (PPARɣ) is a transcription factor that belongs to the superfamily of nuclear receptors. Upon stimulation, PPARɣ forms a heterodimer with the retinoid X receptor, and this heterodimer regulates the transcription of various target genes.^3^ PPARɣ has a large binding pocket that enables it to interact with naturally occurring and synthetic ligands of great structural variety. A group of synthetic, high affinity agonists for PPARɣ are the thiazolidinediones (TZDs), which include pioglitazone and rosiglitazone. Initially, TZDs were developed to reduce insulin resistance and thereby to treat type 2 diabetes.^4^^,^^5^ However, a number of studies also suggested that PPARɣ agonists have direct renoprotective effects in experimental diabetes.^6^, ^7^, ^8^ Moreover, PPARɣ agonists have also been suggested to be renoprotective in several (experimental) non-diabetic glomerular diseases, for example, human focal segmental glomerulosclerosis.^9^, ^10^, ^11^, ^12^, ^13^

A recent study showed that PPARɣ agonists reduced heparanase (HPSE) gene transcription in hepatocellular carcinoma metastases by direct binding of PPARɣ to the HPSE promoter.^14^ HPSE is the only mammalian enzyme that can cleave negatively charged heparan sulfate (HS),^15^ and loss of HS in the GFB has been associated with the development of proteinuria.^16^^,^^17^ We previously showed that HPSE is essential for the development of proteinuria and subsequent renal damage in experimental glomerulonephritis and diabetic nephropathy.^18^^,^^19^ Furthermore, several studies showed reduced proteinuria and improved renal function after inhibition of HPSE activity in glomerular diseases including experimental diabetic nephropathy, glomerulonephritis, anti-GBM antibody disease, and passive Heymann nephritis.^18^^,^^20^, ^21^, ^22^, ^23^

We hypothesized that PPARɣ agonists exert their renoprotective effect by inhibiting the expression of glomerular HPSE since HPSE is essential for the development of proteinuria, TZDs reduce proteinuria, and PPARɣ regulates HPSE expression in liver cancer cells. In the current study, we evaluated the effects of PPARɣ agonism and antagonism on glomerular HPSE and HS expression *in vivo* and *in vitro*. We evaluated whether PPARɣ directly regulates HPSE transcription. Finally, we assessed the plasma HPSE activity in type 2 diabetes mellitus (T2DM) patients before and after 16/24 weeks treatment with the PPARɣ agonist pioglitazone.

## Methods

### Animals

Adriamycin nephropathy (AN) was induced in Wistar rats (8-week-old; Charles River Laboratories, Wilmington, MA (RRID:RGD_2312511)) as previously described.^11^ Rats were treated daily with 12 mg/kg pioglitazone (Sigma–Aldrich) or vehicle via an intraperitoneal injection. After 6 weeks, rats were sacrificed. In addition, healthy Wistar rats were treated with daily intraperitoneal injections with 2.5 mg/kg body weight of the PPARɣ antagonist GW9662 (Sigma–Aldrich) or vehicle, as previously described.^11^ Rats were sacrificed after 3 weeks.

### Cell culture

Opossum Kidney (OK) cells (RRID:CVCL_0472) were cultured as described previously.^24^ Conditionally immortalized mouse podocytes (mPC-5, RRID:CVCL_AS87), human podocytes (hPOD), mouse glomerular endothelial cells (mGEnC-1), and human glomerular endothelial cells (ciGEnC, RRID:CVCL_W185) were cultured as described previously.^25^, ^26^, ^27^, ^28^ HPSE was silenced in mGEnC-1 by a HPSE shRNA construct (Qiagen, Venlo, the Netherlands). Differentiated mPC-5 and mGEnC-1 were stimulated with vehicle or 0.25 μg/ml adriamycin (Sigma–Aldrich) and treated with 10 μM pioglitazone (Sigma–Aldrich). In addition, differentiated mPC-5 and mGEnC-1 were treated with 1 μM or 10 μM of the PPARɣ antagonist GW9662 (Sigma–Aldrich). All experiments were performed at least in triplicate.

### Immunofluorescence staining

Glomerular expression of HPSE and HS was determined by immunofluorescence staining as described previously.^29^ Primary antibodies included the polyclonal anti-HPSE antibody HPA1 (ProsPecTany, Rehovot, Israel (RRID:AB_2246577)) and the VSV-tagged single chain HS variable fragment (scFv) antibody EV3B2 (N-, and 6-O sulfation).^30^ Secondary antibodies included goat anti-rabbit IgG Alexa 488 (Invitrogen Life Technologies, RRID:AB_143165) for detection of HPSE and anti-VSV Cy3 (Sigma–Aldrich, RRID:AB_259043) for detection of EV3B2. Staining intensities of HPSE and HS were scored in fifty glomeruli per section on a scale between 0 (no staining) and 10 (maximal staining intensity). Scoring was performed on blinded sections by two independent investigators.

### RNA isolation and real-time PCR

Total RNA was isolated from rat renal cortex, mPC-5 and mGEnC-1 using the RNeasy mini kit (Qiagen), according to manufacturer's instructions. 1 μg of RNA was reverse transcribed into cDNA using the Transcriptor First Strand cDNA Synthesis kit (Roche Diagnostics, Mannheim, Germany). HPSE mRNA expression was determined by real-time PCR on the CFX real-time PCR system (Bio-Rad Laboratories, Hercules, CA, USA) using SYBR Green Supermix (Roche Diagnostics) and gene-specific primers (Table 1; Isogen Life Science, de Meern, the Netherlands). Relative HPSE mRNA expression was determined using the delta–delta C_T_ method with glyceraldehyde-3-phosphate dehydrogenase (GAPDH) as housekeeping gene.

**Table 1:** Primers used in real-time PCR.

Target gene	Primer sequence
mHPSE	(F) 5′-GAGCGGAGCAAACTCCGAGTGTATC-3′ (R) 5′-GATCCAGAATTTGACCGTTCAGTT-3′
rHPSE	(F) 5′-GAGCGAAGCAAACTCCGAGTGTAC-3′ (R) 5′-GATCGGTTTGACCGTTCAGTTGG-3′
GAPDH	(F) 5′-AGAAACCTGCCAAGTATGATGAC-3′ (R) 5′-TCATTGTCATACCAGGAAATGAG-3′

mHPSE, mouse heparanase; rHPSE, rat heparanase; mGAPDH, glyceraldehyde-3-phosphate dehydrogenase; F, forward; R, reverse.

### Heparanase activity assay

Renal cortical HPSE activity was determined by a commercially available assay (AMS Biotechnology, Abingdon, UK, cat no# Ra001-02-K) following manufacturer's instructions. Plasma HPSE activity was determined by a commercially available heparan degrading enzyme assay kit (Takara Bio, Shiga, Japan, cat no#MK412) according to the manufacturer's instructions.

### Heparanase protein assay

HPSE protein in plasma was measured using a human heparanase ELISA kit (Abcam, Cambridge, UK, cat. no. #ab256401) according to the manufacturers instruction.

### Transendothelial albumin passage

mGEnC-1 were seeded on polyester membranes in tissue culture inserts (Corning Incorporated, NY, USA). After differentiation, cells were treated with adriamycin in the presence or absence of the PPARɣ agonist pioglitazone, or with the PPARɣ antagonist GW9662 as outlined above. Transendothelial albumin passage was determined as described previously.^31^

### Luciferase reporter assay

OK cells transfected with a pGL3 firefly luciferase vector containing the 3.5 kb promoter region of the human HPSE gene^32^ or an empty pGL3 vector construct, were treated with 10 μM GW9662 (Sigma–Aldrich). The pRL-CMV construct (Promega Corp., Fitchburg, WI) was used to correct for transfection efficiency. OK cells were harvested 24 h after transfection and luciferase activity was determined using the Dual-Luciferase reporter assay (Promega) according to the manufacturer's instructions.

### Chromatin immunoprecipitation assay to determine PPARɣ binding to the HPSE promoter

Chromatin was cross-linked using 1.5 mM ethylene glycol bis (succinimidyl succinate) (EGS, Thermofischer Scientific) and 1% formaldehyde (Sigma) in phosphate buffered saline (PBS). Cells were scraped, the cell suspensions were collected and centrifuged at 2500 rpm for 4 min. The pellet, containing the cells was stored at −80 °C until further use. After thawing, nuclei were isolated with subsequent lysis buffer 1 (50 mM Hepes-KOH pH 7.5, 140 mM NaCl, 1 mM EDTA, 10% glycerol, 0.5% NP-40, 0.25% Triton-X-100) supplemented with freshly added proteinase inhibitor cocktail (1:100, v/v), followed by centrifugation at 2000 × *g* for 5 min. Nuclei were dissolved in lysis buffer 2 (10 mM Tris HCl pH 8.0, 200 mM NaCl, 1 mM EDTA, 0.5 mM EGTA) supplemented with freshly added phosphatase inhibitor, followed by centrifugation at 2000 × *g* for 5 min. Afterwards, the pellet was dissolved in lysis buffer 3 (10 mM Tris HCl pH 8.0, 100 mM NaCl, 1 mM EDTA, 0.5 mM EGTA, 0.1% Na-deoxycholate, 0.5% N-lauroylsarcosine, 1% NP-40) supplemented with freshly added phosphatase inhibitor. The chromatin was sonicated using a Bioruptor (Diagenode, Seraing, Belgium) with 10 cycles of 30′ ON/OFF. 1% (v/v) Triton-X-100 was added to the sonicated samples and the samples were centrifuged on full speed at 4 °C for 10 min and the pellet discarded. Immunoprecipitation was performed with 1.07 μg of PPARɣ antibody (Genetex) or IgG control (Abcam ab46540) coupled to 30 μl of Dynabeads Protein-A (Invitrogen). 5 μg of pre-cleared chromatin was added to the antibody–beads complexes and incubated at 4 °C with rotation overnight. Beads were washed with RIPA buffer (50 mM HEPES pH 7.6, 1 mM EDTA, 0.7% Na-deoxycholate, 1% NP-40, 0.5M LiCl) and afterwards with TE buffer (10 mM Tris–HCl, pH8.0, 1 mM EDTA, pH8.0). The antigen–antibody complexes were eluted with elution buffer (1% SDS, 0.1M NaHCO3) at 62 °C for 4 h. Input samples served as controls. Eluted samples were incubated with 2 μl RNAse (10 mg/ml stock, Thermo Scientific) at 37 °C for 1 h. Next, 4 μl of Proteinase K (20 mg/ml stock, Roche Diagnostics, Germany) was added to the samples and incubated at 55 °C for 2 h. DNA fragments were purified using the QIAquick PCR purification kit (Qiagen) according to the manufacturers’ instructions. DNA enrichment was analyzed by real-time PCR using a forward and reverse primer for the predicted PPARɣ-binding site in the promoter of HPSE (Table 2). The enrichment of the promoter sequences in the DNA samples was calculated relative to the percentage of input.

**Table 2:** Primers used for ChIP-qPCR assays.

Genomic region	Primer sequence
mHPSE_-1667	(F) 5′-GGCGAGTTGCTAACAGGAAG-3′ (R) 5′-TCTGGAGCCAGACCTGAGAT-3′
mHPSE_-436	(F) 5′-GTTAAAAGCCCCGGTTGAG-3′ (R) 5′-CAATGCTCGGATCAGGTTTT-3′
mHPSE_+1007	(F) 5′-GTGCCAGTCTGCAAGTGTGT-3′ (R) 5′-TGTACCTCGCATGCAAGAAG-3′

mHPSE_-1667, mouse heparanase 1667 bases before HPSE TSS; mHPSE_-436, mouse heparanase 436 bases before HPSE TSS, mHPSE_+1007, mouse heparanase 1007 bases after HPSE TSS; F, forward; R, reverse.

### Patients and study design

Patient plasma samples (n = 38) were obtained from two separate studies. 34 patient plasma samples were obtained from the PIRAMID (Pioglitazone Influence on tRiglyceride Accumulation in the Myocardium In Diabetes) study, which was a 24-week prospective, randomized, double-blind, double-dummy with active comparator, 2-center parallel-group intervention.^33^ Males 45 to 65-year old with uncomplicated T2DM were eligible. The inclusion criteria were a glycohemoglobin level of 6.5%–8.5% at screening, body mass index [weight/(length^2^)] of 25–32 kg/m^2^, and blood pressure below 150/85 mm Hg. The exclusion criteria were any clinically significant disorder, particularly any history or complaints of cardiovascular or liver disease or diabetes-related complications, and prior use of thiazolidinediones or insulin. Study procedures are described previously.^33^ In short, patients received pioglitazone (15 mg once daily, titrated to 30 mg once daily after 2 weeks) and underwent outcome measurements at baseline and at study termination after 24 weeks. Four patient plasma samples were obtained from a 16-week phase IIIB multicenter randomized double-blind study.^34^^,^^35^ Inclusion and exclusion criteria of this study were similar to the criteria described for the PIRAMID study. Study procedures are described previously.^34^^,^^35^ In short, patients received pioglitazone (30 mg once daily), and outcome measurements were obtained at baseline and at study termination after 16 weeks.

### Statistical analysis

Data is presented as mean ± SEM. Significance was evaluated by a one-way ANOVA and *post hoc* analysis with the Tukey's multiple comparison test. A student's *t*-test was used to evaluate differences between two groups. Significance in transendothelial albumin passage was evaluated by a 2-way repeated measures ANOVA with a Bonferroni post-test. In the PPARɣ-ChIP, outliers were identified using a ROUT-test with a Q-value of 5%. Significant differences were evaluated by a paired student's *t*-test. For the patient data, D'Agostino & Pearson normality test was performed to test for normality of data. Significance was determined by Student's t-test or Mann Whitney test to compare two groups. Relationship analysis was performed using Pearson's correlation coefficient. All analysis were performed using GraphPad Prism V.8.4.2 (La Jolla, USA). A P-value of ≤0.05 was considered statistically significant.

### Ethics statement

All animal experiments were approved by the Animal Ethical Committee of the Radboud University Nijmegen and performed in accordance with the guidelines of the Dutch Council for Animal Care (Approval number DEC2014136). The protocols for the patient studies were approved by the medical ethics committee at each study site (Approval numbers P04.193 and NL2264902908CCMO), and the study was performed in full compliance with the Declaration of Helsinki. Written informed consent was obtained from all participants.

### Role of funders

The funding sources had no role in study design, data collection, data analysis, interpretation and writing of this manuscript.

## Results

### Adriamycin-induced heparanase expression is attenuated by the PPARɣ agonist pioglitazone

**Fig. 1:**
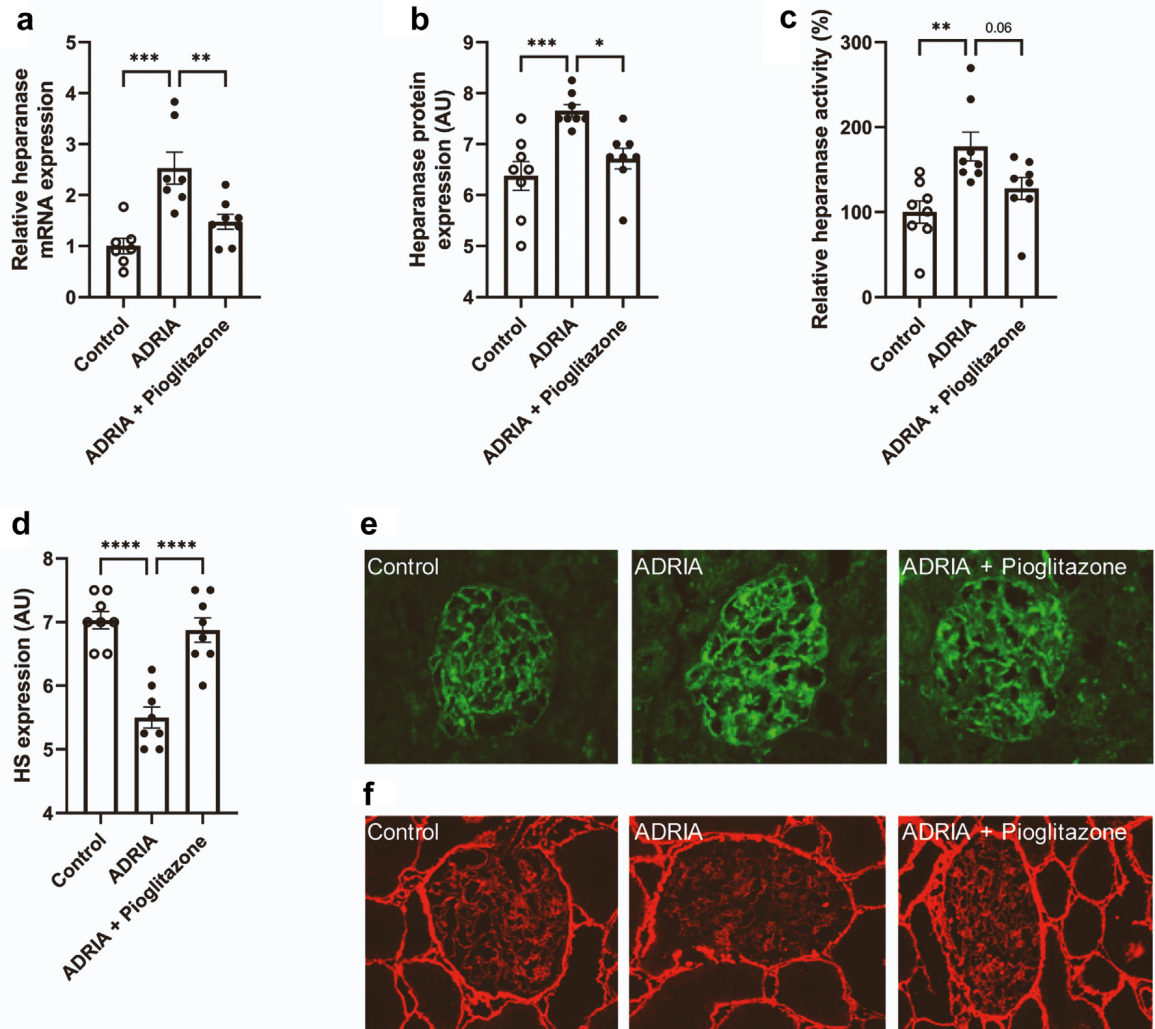
**The PPARɣ agonist pioglitazone reduced glomerular heparanase expression and preserved glomerular HS expression in rats with AN.** (**a**) Cortical heparanase mRNA expression, (**b** and **e**) glomerular heparanase protein expression by quantification of immunofluorescence images, and (**c**) cortical heparanase activity were significantly increased 6 weeks after induction of AN. Heparanase expression and activity were reduced by daily treatment with 12 mg/kg body weight pioglitazone. (**d** and **f**) Glomerular HS expression was significantly reduced by induction of AN, but preserved by daily treatment with pioglitazone. Representative pictures of (**e**) glomerular heparanase protein expression and (**f**) glomerular HS expression (magnification ×400). 8 rats per group were used for analyses. Data are expressed as mean ± SEM. ∗P < 0.05, ∗∗P < 0.01, ∗∗∗P < 0.001, and ∗∗∗∗P < 0.0001. AN, adriamycin nephropathy; ADRIA, adriamycin; AU, arbitrary units.

To study the effect of PPARɣ agonism on glomerular HPSE and HS expression *in vivo*, adriamycin nephropathy (AN; an animal model for human FSGS) was induced in rats that were subsequently treated with the PPARɣ agonist pioglitazone or vehicle for 6 weeks. Induction of AN resulted in the development of proteinuria, which was significantly reduced by treatment with pioglitazone, as we described previously.^11^ Cortical HPSE mRNA expression (Fig. 1a), glomerular HPSE protein expression (Fig. 1b and e), and cortical HPSE activity (Fig. 1c) were significantly increased by induction of AN. Daily treatment with pioglitazone normalized HPSE expression and activity (Fig. 1a–c and e). Moreover, glomerular HS expression was significantly reduced by induction of AN but preserved by treatment with pioglitazone (Fig. 1d and f).

### The PPARɣ agonist pioglitazone reduced HPSE expression and transendothelial albumin passage *in vitro*

**Fig. 2:**
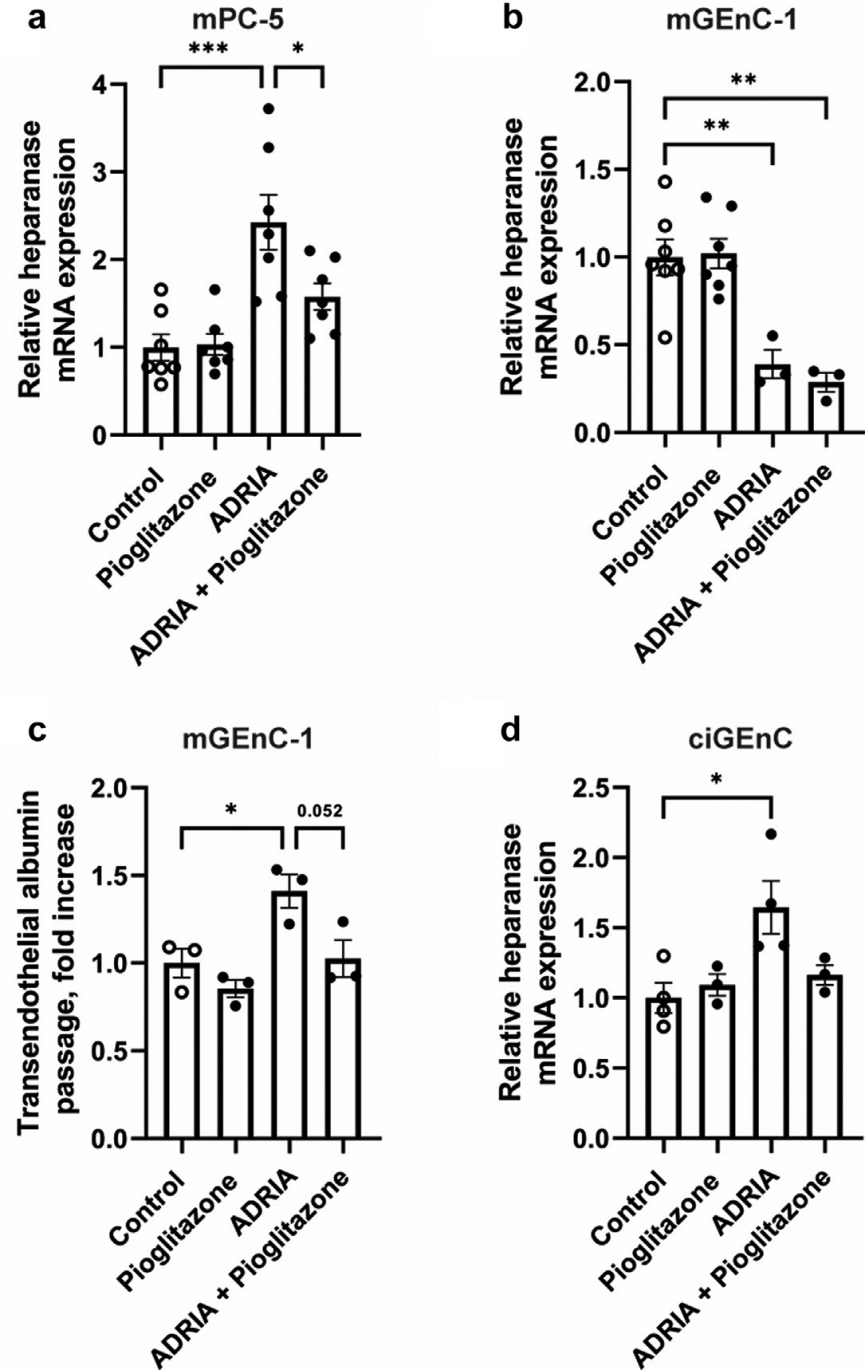
**The PPARɣ agonist pioglitazone reduced heparanase expression and transendothelial albumin passage *in vitro*.** (**a**) Stimulation of mouse podocytes (mPC-5) with adriamycin for 24 h significantly increased heparanase mRNA expression. Heparanase mRNA expression was reduced by treatment with 10 μM of the PPARɣ agonist pioglitazone. (**b**) Stimulation of mouse glomerular endothelial cells (mGEnC-1) with adriamycin for 16 h reduced heparanase mRNA expression. Heparanase mRNA expression was not affected by treatment with 10 μM of the PPARɣ agonist pioglitazone. (**c**) Stimulation of mGEnC-1 with adriamycin for 16 h significantly increased the passage of albumin across the endothelial monolayer. Transendothelial albumin passage was reduced by treatment with 10 μM of the PPARɣ agonist pioglitazone. (**d**) Stimulation of human glomerular endothelial cells (ciGEnC) with adriamycin for 24 h increased heparanase mRNA expression. Heparanase mRNA expression was ameliorated by treatment with 10 μM of the PPARɣ agonist pioglitazone. Data are expressed as mean ± SEM. ∗P < 0.05, ∗∗P < 0.01, and ∗∗∗P < 0.001. ADRIA, adriamycin.

To extend the *in vivo* effects of the PPARɣ agonist pioglitazone on glomerular HPSE expression in AN, we evaluated whether pioglitazone regulates HPSE expression in cultured mGEnC-1, mPC-5, ciGEnC and hPOD. Cell stimulation with adriamycin significantly induced HPSE mRNA expression in mouse podocytes, whereas HPSE mRNA expression was reduced by treatment with pioglitazone (Fig. 2a). Stimulation of mouse glomerular endothelial cells with adriamycin reduced HPSE mRNA expression, which was not affected by treatment with pioglitazone (Fig. 2b). Notably, HS expression on mGEnC-1 was reduced by stimulation with adriamycin.^31^ At the functional level, transendothelial albumin passage was significantly increased by stimulation with adriamycin, which was reduced by additional treatment with pioglitazone (Fig. 2c). Human glomerular endothelial cells did upregulate HPSE mRNA expression upon adriamycin stimulation, which was ameliorated by treatment with pioglitazone (Fig. 2d). On the contrary, human podocytes show no response to adriamycin on HPSE mRNA expression, which might indicate that hPODs are not a good model system for this study (data not shown).

### Pharmacologic inhibition of PPARɣ induces heparanase expression and activity *in vivo*

**Fig. 3:**
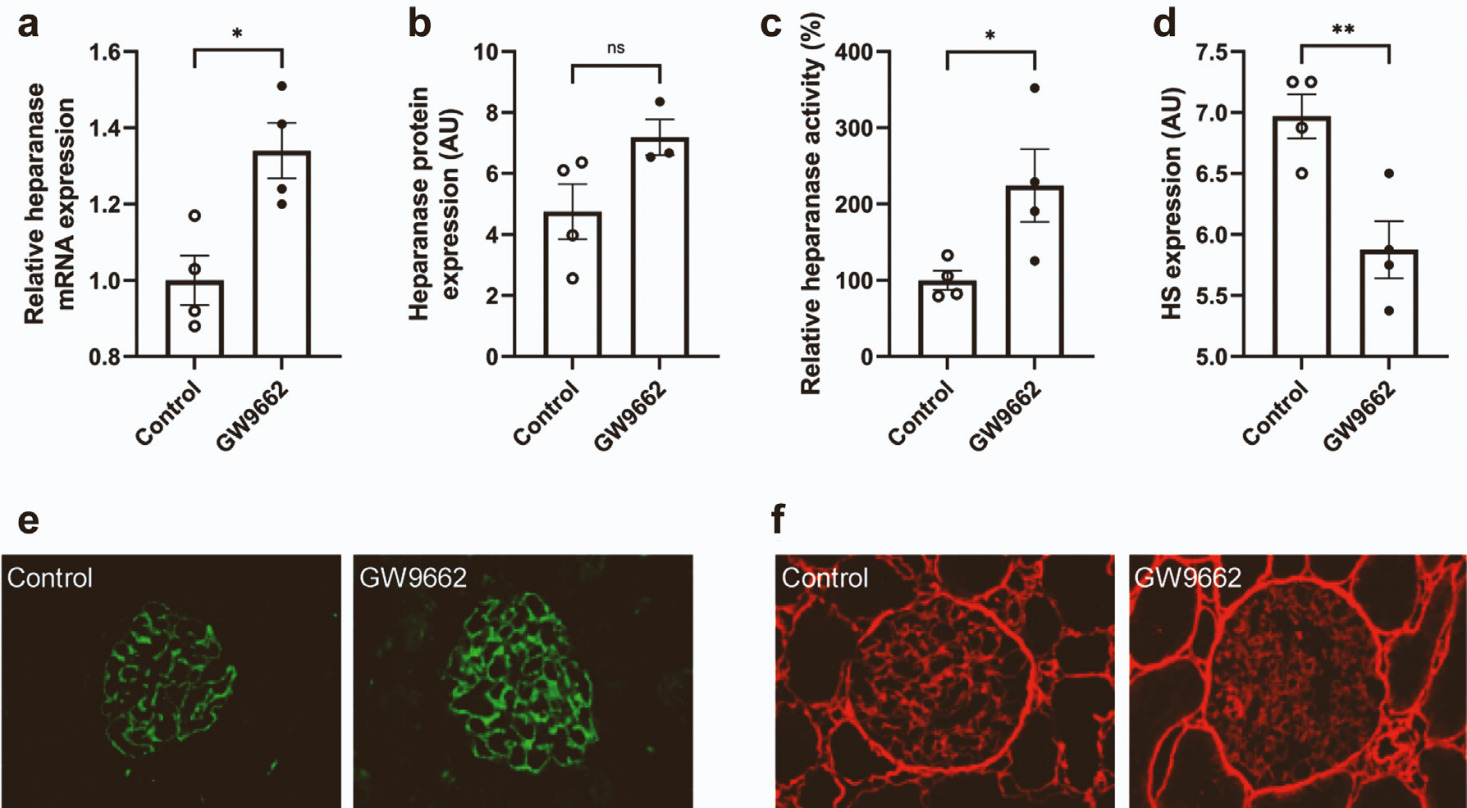
**The PPARɣ antagonist GW9662 induced glomerular heparanase expression and reduced glomerular HS expression in mice.** (**a**) Cortical heparanase mRNA expression, (**b** and **e**) glomerular heparanase protein expression, and (**c**) cortical heparanase activity were increased by treatment with 2.5 mg/kg body weight of the PPARɣ antagonist GW9662 for 3 weeks, whereas glomerular HS expression was reduced according to semi-quantitative analysis of immunofluorescence images (**d** and **f**). Representative pictures of (**e**) glomerular heparanase protein expression and (**f**) glomerular HS expression (magnification ×400). 4 rats per group were used for analysis. Data are expressed as mean ± SEM. ∗P < 0.05 and ∗∗P < 0.01. AU, arbitrary units.

Healthy rats were treated daily with the irreversible PPARɣ antagonist GW9662 for 3 weeks to study the effects of pharmacologic inhibition of PPARɣ on glomerular HPSE and HS expression *in vivo*. As we described previously, treatment with GW9662 induced significant proteinuria.^11^ Cortical HPSE mRNA expression (Fig. 3a), glomerular HPSE protein expression (Fig. 3b and e), and cortical HPSE activity (Fig. 3c) were increased by treatment with GW9662, although this was not significant for HPSE protein expression (P = 0.09). In line, glomerular HS expression was reduced by treatment with GW9662 (Fig. 3d and f).

### The PPARɣ antagonist GW9662 induces transendothelial albumin passage *in vitro* in a heparanase-dependent manner

**Fig. 4:**
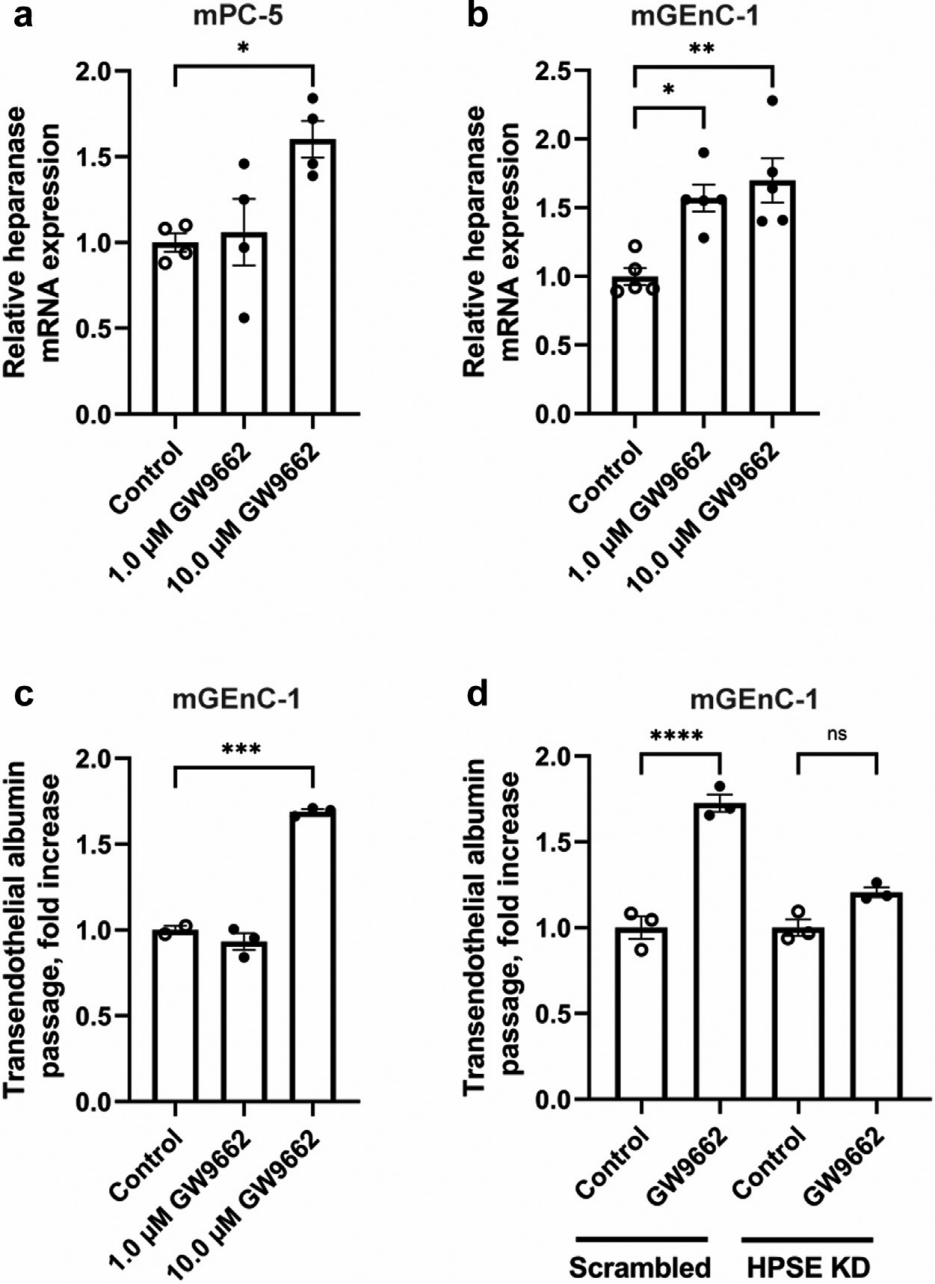
**The PPARɣ antagonist GW9662 induced heparanase expression and increased transendothelial albumin passage in a heparanase-dependent manner.** (**a**) Treatment of mouse podocytes with 10 μM of the PPARɣ antagonist GW9662 for 24 h significantly increased heparanase mRNA expression. (**b**) Treatment of mouse glomerular endothelial cells (mGEnC-1) with 1 μM and 10 μM of the PPARɣ antagonist GW9662 for 24 h resulted in increased heparanase mRNA expression. (**c**) The cumulative passage of albumin across mGEnC-1 monolayers was significantly increased by treatment with 10 μM of the PPARɣ antagonist GW9662 for 24 h. (**d**) Treatment of heparanase-silenced mGEnC-1 (knockdown efficiency 68%) with 10 μM of the PPARɣ antagonist GW9662 for 24 h led to a lower transendothelial albumin passage compared with scrambled mGEnC-1 treated with GW9662. Data are expressed as mean ± SEM. ∗P < 0.05, ∗∗P < 0.01, ∗∗∗P < 0.001, and ∗∗∗∗P < 0.0001.

Cultured mGEnC-1, mPC-5, ciGEnC and hPOD were treated with 1 or 10 μM of the PPARɣ antagonist GW9662 for 24 h to evaluate the effects of PPARɣ antagonism on glomerular HPSE expression *in vitro*. Treatment of mouse podocytes with 10 μm GW9662 significantly increased HPSE mRNA expression, whereas treatment with 1 μM GW9662 was not effective (Fig. 4a). HPSE mRNA expression in mGEnC-1 was increased by treatment with both 1 and 10 μM GW9662 (Fig. 4b). At the functional level, passage of albumin across an mGEnC-1 monolayer was significantly increased by treatment with 10 μM GW9662 (Fig. 4c). On the contrary, human podocytes and endothelial cells did not up-regulate HPSE mRNA expression upon stimulation with GW9662 (data not shown).

To evaluate whether the GW9662-induced transendothelial albumin passage was HPSE-dependent, HPSE was silenced in mGEnC-1, which resulted in ∼50% reduction of HPSE mRNA expression. The GW9662-induced increase in transendothelial albumin passage was significantly ameliorated in HPSE-silenced mGEnC-1 compared with mGEnC-1 transfected with a scrambled shRNA (Fig. 4d), indicating that the GW9662-induced transendothelial albumin passage is HPSE-dependent.

### The PPARɣ antagonist GW9662 increased heparanase promoter activity

**Fig. 5:**
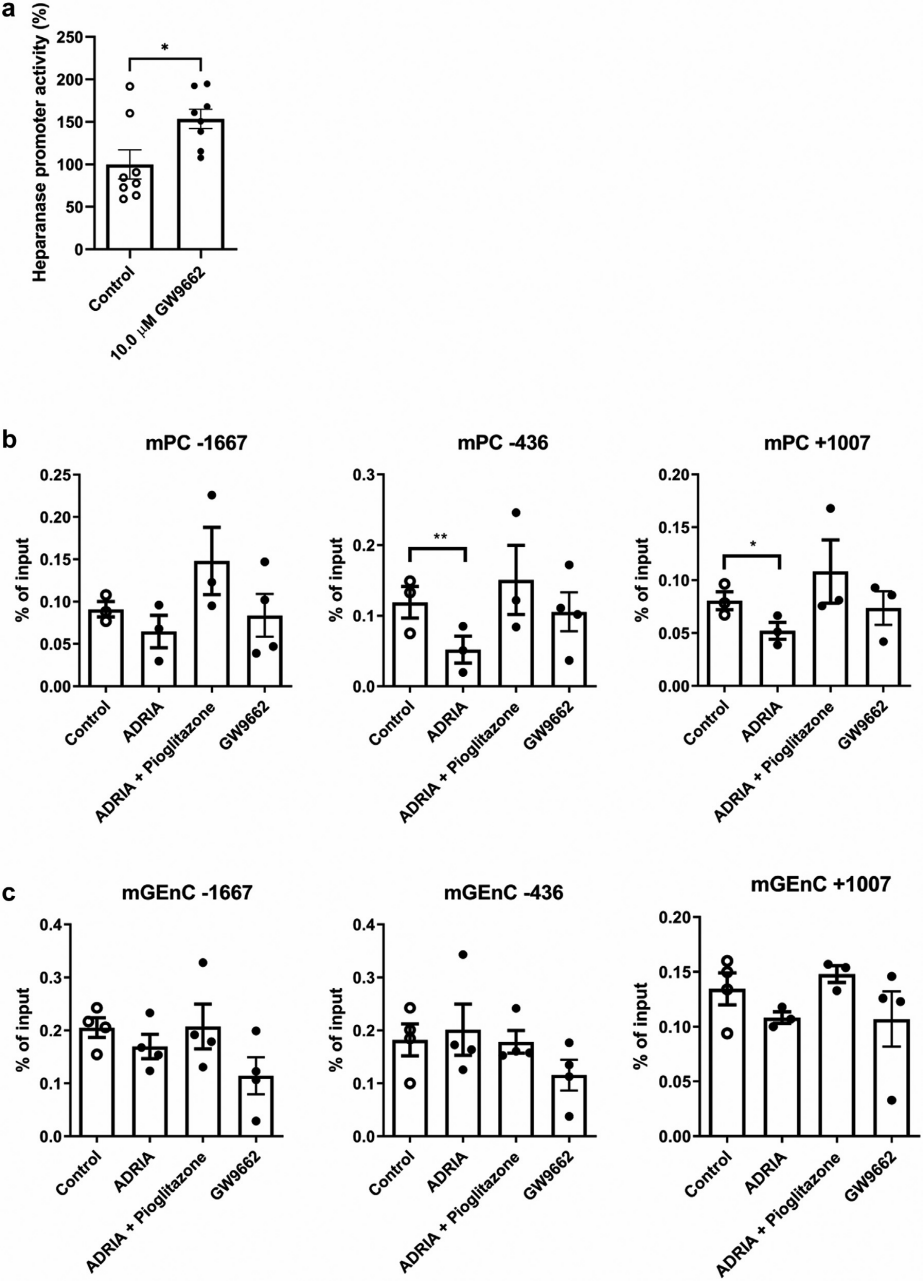
**PPARɣ directly binds to the heparanase promoter.** (**a**) Opossum kidney cells were transfected with the HPR1-3.5 HPSE promoter construct and treated with 10 μM of the PPARɣ antagonist GW9662 for 24 h. Treatment with the PPARɣ antagonist GW9662 significantly increased heparanase promoter activity. (**b**) Treatment of mouse podocytes (mPC-5) with adriamycin for 24 h decreased binding of PPARɣ to the heparanase promoter significantly at binding sites −436 and +1007. A trend was observed for binding site −1667. Treatment with 10 μM of the PPARɣ agonist pioglitazone prevented the adriamycin-induced decrease in PPARɣ binding at all binding sites. Treatment with 10 μM of the PPARɣ antagonist GW9662 tended to reduce PPARɣ binding to binding sites −436 and −1667 (**c**) Stimulation of mouse glomerular endothelial cells (mGEnC-1) with adriamycin and/or 10 μM pioglitazone, or GW9662 did not significantly affect PPARɣ binding to the heparanase promoter, although similar trends were observed as found for the mPC-5 cells. Data are expressed as mean ± SEM. ∗P < 0.05, ∗∗P < 0.01.

A luciferase reporter assay was performed to evaluate whether the *in vivo* and *in vitro* observed effects of the PPARɣ antagonist GW9662 on HPSE expression are caused by a direct regulation of HPSE promoter activity.^32^ Treatment with 10 μM GW9662 significantly induced HPSE promoter activity (Fig. 5a).

### Direct binding of PPARɣ to the HPSE promoter is regulated by adriamycin and PPARɣ agonists and antagonists

Next, a Chromatin Immunoprecipitation (ChIP) assay was performed to evaluate whether the observed effects of adriamycin, PPARɣ agonist pioglitazone, and PPARɣ antagonist GW9662 on HPSE expression in mPC-5 and mGEnC-1 cells were mediated by direct binding of PPARɣ to the endogenous HPSE promoter. Within the region of 2000 bases before and after the HPSE transcription start site (TSS), three putative PPARɣ binding sites (PPRE's) can be identified using MotifMap,^36^ i.e at a distance of −1667, −436, and +1007 bases. Binding of PPARɣ to the HPSE promoter was evaluated in cultured mPC-5 and mGEnC-1 treated with adriamycin either with or without pioglitazone, or with the PPARɣ antagonist GW9662, and in untreated cells. In mPC-5 exposed to adriamycin, binding of PPARɣ to the HPSE promoter was significantly reduced for binding site −436 and +1007, whereas there was a similar trend for the binding site −1667. Treatment with pioglitazone prevented the adriamycin-induced decline in PPARɣ binding to all 3 PPARɣ binding sites in the HPSE promoter. However, treatment with GW9662 did not reveal a significant reduction in PPARɣ binding to the PPARɣ binding sites in the HPSE promoter, although there was a trend for lower PPARɣ binding for binding sites −436 and −1667 (Fig. 5b). In mGEnC-1, the different treatments did not significantly change PPARɣ binding to the HPSE promoter for all 3 binding sites. Nevertheless, there is a trend for adriamycin-induced lowering of PPARɣ binding for binding sites −1667 and +1007, as well as for GW9662-induced lowering of PPARɣ binding for all three binding sites (Fig. 5c).

### Pioglitazonde tends to reduce the plasma HPSE activity in type 2 diabetes mellitus patients

Full clinical characterization is described in the original manuscripts of the studies from which the samples have been obtained.^33^, ^34^, ^35^ The most important clinical characteristics of the patients are summarized in Table 3. Overall, hemoglobin A1c (HbA_1C_), plasma glucose, and diastolic blood pressure levels were decreased upon 16/24 weeks pioglitazone treatment. HDL cholesterol was increased after treatment with pioglitazone. The plasma creatinine level, insulin level, LDL cholesterol level, triglycerides level, systolic blood pressure and heart rate remained unaltered.

**Table 3:** Clinical characteristics of patients.

Variables	Baseline	16/24 weeks	P
Number	37	31	
HbA_1C_, %	7.13 (0.19)	6.51 (0.17)	0.023
Plasma creatinine, μmol/L	79.41 (2.37)	82.61 (2.30)	0.153
Plasma glucose, mmol/L	9.08 (0.35)	7.93 (0.29)	0.024
Insulin, ρmol/L	68.16 (5.80)	55.41 (4.29)	0.180
LDL cholesterol, mmol/L	2.61 (0.11)	2.68 (0.81)^a^	0.781
HDL cholesterol, mmol/L	1.12 (0.04)	1.26 (0.05)	0.034
Triglycerides, mmol/L	1.84 (0.16)	1.69 (0.20)	0.412
Systolic blood pressure, mm Hg	131.2 (2.05)	132.1 (1.98)	0.716
Diastolic blood pressure, mm Hg	77.92 (1.19)	81.13 (1.43)	0.041
Heart rate, beats/min	65.14 (1.45)	62.58 (0.98)	0.236

Data are presented as mean (SEM).

HbA_1C_, hemoglobin A1c; LDL, low-density lipoprotein; HDL, high-density lipoprotein.

a Indicates missing value.

**Fig. 6:**
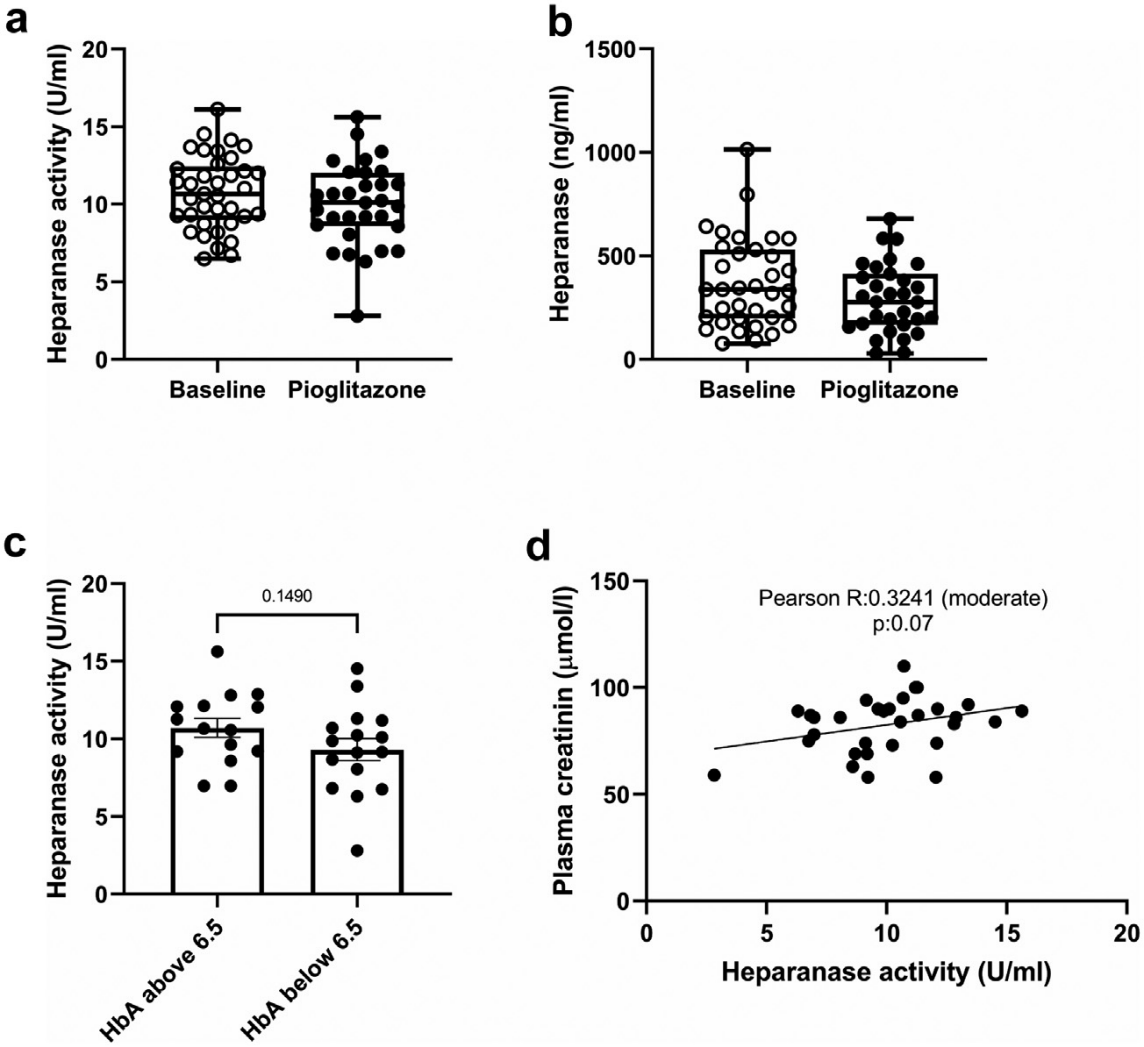
**Plasma heparanase activity before and after treatment with pioglitazone in type 2 diabetes mellitus patients.** (**a**) Plasma heparanase activity of 38 type 2 diabetes mellitus patients before and after 16/24 weeks treatment with pioglitazone depicted as individual values in a box plot to show the difference per quartile. (**b**) Plasma heparanase expression levels of 38 type 2 diabetes mellitus patients before and after 16/24 weeks treatment with pioglitazone depicted as individual values in a box plot to show the difference per quartile. (**c**) Plasma heparanase activity categorised according to the presence of HbA_1C_ above or below 6.5% upon treatment with pioglitazone for 16/24 weeks. (**d**) Correlation between plasma heparanase activity and creatinine levels after 16/24 weeks of treatment with pioglitazone (n = 31). Data are expressed as mean ± SEM.

Patients with type 2 diabetes mellitus (T2DM) are known to have increased levels of HPSE activity, and it is well known that HPSE is essential for the development of diabetic nephropathy.^18^^,^^37^ Therefore, the plasma HPSE activity of T2DM patients was measured at baseline and compared with the HPSE activity level upon 16/24 weeks treatment with pioglitazone. Pioglitazone did not affect the plasma HPSE activity of the T2DM patients (P = 0.21) (Fig. 6a). However, a decrease in plasma HPSE activity can be observed in patients in the lowest quartile of the cohort, which can be appreciated when the lowest quartile of the baseline is compared with the lowest quartile after treatment with pioglitazone presented in the boxplot (P = 0.07) (Fig. 6a). It should be noted that the HPSE activity was measured in plasma, whereas a more pronounced effect of pioglitazone on HPSE activity might be observed in renal cortex. Similarly, a trend of decreased heparanase expression levels in plasma can be observed in the patient cohort after treatment with pioglitazone (P = 0.12) (Fig. 6b). One of the outcome parameters that was reported in the PIRAMID study to change significantly upon pioglitazone treatment is HbA_1C_,^33^ which describes the average blood glucose levels for the last two to three months. We observed higher HPSE activity levels in patients whose HbA_1C_ levels exceeded 6.5% (Fig. 6c), although no direct correlation could be observed between HPSE activity and HbA_1C_ (data not shown). HPSE activity did show a moderate, near significant correlation with plasma creatinine levels (Fig. 6d).

## Discussion

In this study, we showed that PPARɣ regulates the expression of HPSE in cultured podocytes, cultured glomerular endothelial cells, healthy rats and in a rat model for human FSGS. In agreement with our previous studies, rats showed increased glomerular HPSE expression and reduced glomerular HS expression after induction of AN.^31^^,^^38^ Treatment with the PPARɣ agonist pioglitazone normalized glomerular HPSE and HS expression, whereas PPARɣ antagonism induced glomerular HPSE expression and reduced glomerular HS expression. Importantly, in line with the induction of proteinuria *in vivo*, we showed that PPARɣ antagonism induced transendothelial albumin passage *in vitro* in a HPSE-dependent manner. In addition, we showed that the PPARɣ antagonist GW9662 induced HPSE promoter activity, suggesting that the transcription factor PPARɣ directly suppresses HPSE transcription.

The present study shows that PPARɣ is a negative regulator, i.e. repressor, of glomerular HPSE. A previous study revealed that PPARɣ inhibited hepatocellular carcinoma migration by downregulating pro-metastatic genes, including HPSE by direct binding to the HPSE promoter region.^14^ We now show direct binding of PPARɣ to the HPSE promoter in mouse podocytes and glomerular endothelial cells. Adriamycin treatment significantly reduced the binding of PPARɣ to the HPSE promoter in podocytes, and a similar trend was observed for glomerular endothelial cells. PPARɣ agonism inhibits the decline in PPARɣ binding to the HPSE promoter upon adriamycin treatment. In addition, treatment of podocytes and glomerular endothelial cells with the PPARɣ antagonist GW9662 tended to reduce PPARɣ binding to the HPSE promoter. These data suggest that in podocytes and most likely also in glomerular endothelial cells, PPARɣ represses gene expression potentially through co-repressors such as the nuclear receptor corepressor (NCoR) or the silencing mediator of retinoid and thyroid hormone receptors (SMRT) via histone deacetylases and subsequent transcriptional repression of HPSE.^39^

Our *in vitro* results support the hypothesis that PPARɣ regulates HPSE gene expression. Although both mPC-5 and ciGEnC increased HPSE mRNA expression upon treatment with adriamycin, the HPSE mRNA expression from mGEnC-1 decreased upon adriamycin treatment after 16 h. Previously, we observed that the initial response of mGEnC-1 to adriamycin is an increased HPSE mRNA expression in the first hours after exposure, whereas HPSE mRNA expression is decreased after 16 h. However, the HS expression by mGEnC-1 is still decreased after 16 h of adriamycin treatment, corresponding to an increased albumin passage through mGEnC-1 monolayers.

PPARɣ agonists, like the TZDs pioglitazone and rosiglitazone, have already been clinically applied for a few decades. As they reduce insulin resistance, they are mainly used for the treatment of patients with type 2 diabetes.^4^^,^^5^ However, a number of studies also suggest that PPARɣ agonists have direct renoprotective effects.^6^^,^^7^^,^^12^^,^^13^ HPSE has been shown to be essential for the development of diabetic nephropathy, and type 2 diabetes patients show increased levels of HPSE activity.^18^^,^^37^ We showed that plasma HPSE activity in these pioglitazone treated patients was related to their HbA1c level and correlating with serum creatinine levels. Even though no clear effect on plasma HPSE activity could be observed upon pioglitazone treatment, a decrease of HPSE activity in the renal cortex cannot be omitted. Since treatment with pioglitazone and rosiglitazone both reduced proteinuria in patients with non-diabetic renal diseases,^12^^,^^13^^,^^40^ TZDs could be considered for treatment of proteinuria in non-diabetic renal diseases as well. A major drawback of TZDs is the development of severe side effects, such as fluid retention, edema formation, cardiac failure and an increased risk for bone fractures.^41^ With this study, we provide further mechanistic *in vitro* and rat *in vivo* evidence that TZDs have direct renoprotective effects, by repressing glomerular HPSE expression and activity.

In addition to HPSE, several mechanisms have been described that may explain the renoprotective effects of PPARɣ agonists. A previous study showed that the PPARɣ agonist rosiglitazone partially reduced proteinuria by preserving the expression of the slit diaphragm proteins nephrin, podocin, and CD2AP in rats with AN.^9^ More recently, we showed that PPARɣ agonists reduced the expression of the slit diaphragm protein transient receptor potential channel C6 (TRPC6) in podocytes.^11^ Previous studies showed that glomerular TRPC6 expression is increased in several proteinuric diseases, suggesting that TRPC6 plays a role in the development of proteinuric diseases.^42^ PPARɣ negatively regulated TRPC6 expression by direct binding to the TRPC6 promoter, and thereby reduced podocyte damage and the development of proteinuria in rats with AN.^11^ In addition to the slit diaphragm proteins TRPC6, nephrin, podocin and CD2AP, PPARɣ also reduced the expression of transforming growth factor-β (TGF-β), endothelin-1 and the renin-angiotensin-aldosterone system (RAAS), and increased the bioavailability of nitric oxide.^43^, ^44^, ^45^, ^46^, ^47^, ^48^ Interestingly, all these aforementioned factors are involved in the regulation of HPSE as well.^38^^,^^49^, ^50^, ^51^ Together, there appear to be several mechanisms underlying the renoprotective effects of PPARɣ agonists.

There are also some limitations in our study. First, the patient samples are from T2DM patients, while pioglitazone is known to have a glucose-lowering effect and lower blood glucose level may affect HPSE expression and/or activity as well.^52^^,^^53^ However, the animal model that was used in this study was not a diabetes model. Second, we only assessed HPSE expression levels but not the more relevant HPSE activity for the *in vitro* models because we were unable to measure this for the endothelial cells. Third, the route of administration of pioglitazone can be discussed; we have administered pioglitazone via i.p injection. Although pioglitazone is administered orally in patients, others have administered pioglitazone via i.p injection in animal studies as well.^54^ Fourth, the use of cell lines in mono-culture is not ideal, since it is well known that the cross-talk between podocytes and endothelial cells play a key role in disease manifestation.^55^, ^56^, ^57^ Future experiments should thus focus on the development of co-cultures or organ-on-a-chip technologies able to test the effects of e.g. PPARy agonism on such interactions. Finally, we only focused on the glomerular cells as possible source and target of HPSE, whereas it is well known that various cell-types, including non-glomerular endothelial cells and immune cells, can be a source of HPSE and various cell types and the extracellular matrix can be a target of HPSE.^53^^,^^58^

Our current study provides an additional mechanism for the renoprotective effects of pioglitazone and other PPARɣ agonists. By reducing glomerular HPSE expression and activity, glomerular HS expression is preserved and the development of proteinuria is prevented. In the past, AN was mainly regarded as a podocyte damage model for human FSGS. However, more recent studies showed that glomerular endothelial cells play a crucial role in the development of AN, as the glomerular endothelial glycocalyx thickness is reduced by 80% in mice with AN, and HS expression on cultured mouse glomerular endothelial cells is reduced by Adriamycin.^31^^,^^59^ Additionally, a recent study showed that endothelial cell damage precedes podocyte damage in AN,^60^ further highlighting the importance of the glomerular endothelium in the development of AN. Our current data suggest that PPARɣ agonists have direct protective effects on both glomerular endothelial cells and podocytes, by reducing HPSE expression and transendothelial albumin passage.

In conclusion, our study suggests that PPARɣ agonists like pioglitazone reduce proteinuria by inhibiting glomerular HPSE expression, thereby providing an additional mechanism explaining the anti-proteinuric and renoprotective effects of thiazolidinediones in clinical practice.

## Contributors

TN, GK and JvdV designed the study; TN, GK, JAAMK, JLH, TR and JvdV obtained funding and supervised the study; THvK provided crucial reagents; MG, BB, MS, LG, RS, and MdG carried out experiments; MG, BB, MS, LG, and MMH drafted the paper and the figures; MG, MS, LG, BB, TN, GK and JvdV accessed and verified the data; JvdB, JAAMK, DHvR, RWvdM and HJL provided the patient materials. All authors revised and approved the final version of the manuscript.

## Data sharing statement

This study did not generate any new unique reagents, datasets or code. The main data supporting the findings of this study are available within the paper. Further information and requests for resources and reagents should be directed to the corresponding author.

## Declaration of interests

The authors declare that there are no conflicts of interest.
